# Incidence and risk factors of blowout within 90 days after a primary Hartmann’s procedure: a retrospective cohort study

**DOI:** 10.1007/s00423-023-02967-5

**Published:** 2023-07-14

**Authors:** Josefine Secher, Rogini Balachandran, Lene Hjerrild Iversen

**Affiliations:** 1 Department of Surgery, Aarhus University Hospital, Palle Juul-Jensens Boulevard 99, Aarhus, Denmark; 2 Department of Clinical Medicine, Aarhus University, Aarhus, Denmark

**Keywords:** Hartmann, Blowout, Emergency, Cancer, Diverticulitis, Pelvic abscess

## Abstract

**Purpose:**

The literature reports a varying occurrence (3–33%) of blowout of the rectal remnant after Hartmann’s procedure, and there is a lack of multivariate analyses on potential risk factors for blowout following Hartmann’s procedure. We aimed to estimate the incidence of blowout within 90 days after a primary Hartmann’s procedure and to identify potential risk factors for blowout through multivariate analysis.

**Methods:**

A retrospective cohort study was conducted at the Department of Surgery, Aarhus University Hospital, a Danish primary and tertiary hospital. Patients who underwent primary surgery with Hartmann’s procedure irrespective of surgical setting and indications between September 2016 and August 2021 were included. Blowout was defined as a defective closure line of the rectal stump or a pelvic abscess.

**Results:**

A total of 178 patients were included, and blowout occurred in 30 patients (16.9%) within 90 days after a primary Hartmann’s procedure. Multivariate analysis showed increased risk of blowout among patients with Hinchey IV diverticulitis (relative risk 6.32 (95% CI 4.09–9.75)), previous radiotherapy (relative risk 3.35 (95% CI 1.67–6.74)), and alcohol overconsumption (relative risk 1.69 (95% CI 1.05–2.72)). Intraoperative insertion of a Foley catheter in the rectal remnant significantly reduced the risk of blowout within 90 days after a primary Hartmann’s procedure (relative risk 0.18 (95% CI 0.05–0.65)).

**Conclusion:**

Blowout remains a severe and common complication within 90 days after a primary Hartmann’s procedure. Hinchey IV diverticulitis, pelvic radiotherapy, and alcohol overconsumption are risk factors. An intraoperatively inserted rectal Foley catheter is a protective factor and can be considered used in all patients undergoing Hartmann’s procedure.

**Supplementary Information:**

The online version contains supplementary material available at 10.1007/s00423-023-02967-5.

## Introduction

Hartmann’s procedure (HP) is a surgical procedure for removing pathology in the rectosigmoid colon leaving the patient with an end-colostomy and the rectal remnant sealed by stapling or hand suturing [1]. HP is primarily considered in patients in whom a primary colorectal anastomosis is deemed unsafe, undesired, or unfeasible. Mainly, it is used in emergency cases like bowel obstruction, bowel perforation (e.g., perforated diverticulitis), volvulus, bowel ischemia, postoperative complications, and trauma [2–4], all of which are most often associated with septicemia and inflammation. HP also plays a role in the elective setting for indications like colorectal cancer (CRC), colonic stenosis, multivisceral resections, impaired sphincter function, and, especially in patients with moderate to severe comorbidities, frailty and other conditions associated with an increased risk of anastomotic leakage [5–7]. Restoration of bowel continuity can be considered subsequently [1].

HP has over time been considered a procedure with low morbidity and few post-operative complications [5, 8] as compared to surgery where a primary anastomosis is established. However, up to 40.6–53.3% of patients develop postoperative complications with abdominal abscess, wound infection, and sepsis being the most frequent [9, 10]. Pelvic abscesses resulting in pelvic sepsis is a serious complication; however, existing literature regarding pelvic abscesses after HP is conflicting and the occurrence is reported in the range from 3.0–6.4% [8, 11] up to 11–32.9% [12–15]. The abscess formation is presumably caused by rectal blowout secondary to surgical wound dehiscence, i.e., leakage of the rectal remnant. Often, this is characterized by formation of pus in relation to the rectal stump, manifested by abscess formation or rectal discharge of pus [12]. The pathophysiology of blowout following HP is thus to be compared to anastomotic leakage following surgery with construction of an anastomosis [9].

Previously reported potential risk factors for formation of a pelvic abscess following HP include male sex, lack of foot pulses (indicating poor iliac/lower extremity circulation), transection at a low level of the rectum [12], and preoperative radiotherapy [11]. Few studies have evaluated pelvic abscesses and blowout following HP as a combined primary outcome, and few have investigated potential risk factors for blowout. Most studies have investigated selected cohorts with exclusively specific indications for HP, while some are small-scale studies or studies based on surgery performed more than 10 years ago [4, 16, 17]. To our knowledge, potential risk factors for blowout after HP have only been examined in univariate analyses.

Therefore, we evaluated rectal blowout and pelvic abscesses following primary surgery with HP in a contemporary setting and regardless of the indication. The primary aim was to evaluate the incidence of blowout within 90 days after primary HP, and the secondary aims were to identify possible risk factors for blowout through multivariate analysis, as well as to estimate length of hospital stay (LOS), readmission rate, and 30- and 90-day mortality rates for patients undergoing primary HP.

## Materials and methods

### Study design and setting

We conducted a retrospective cohort study at the Department of Surgery, Aarhus University Hospital, Denmark. Our institution is a primary and tertiary referral center for colorectal diseases in the Central Denmark Region with approximately 1.3 million inhabitants. It is also a national referral center for locally advanced and recurrent rectal cancer, peritoneal metastases treated with cytoreductive surgery and hyperthermic intraperitoneal chemotherapy (HIPEC), among others, for all five regions of Denmark. Aarhus University Hospital is part of the tax-supported healthcare system in Denmark providing equal rights to free of charge healthcare for all Danish citizens.

### Cohort

All patients, both acute and elective, who had undergone sigmoid or rectal resection with an end-colostomy and closure of the rectal remnant at the Department of Surgery, Aarhus University Hospital, between September 2016 and August 2021 were identified through a regional hospital data information database. Surgical procedures have been recorded since 1996 according to the NOMESCO (Nordic Medico-Statistical Committee) Classification of surgical procedures. We used the operative codes KJFB60 and KJFB61 for sigmoid resection and KJGB10 and KJGB11 for rectal resection. Patients undergoing secondary surgery with HP due to post-operative complications from a primary surgery were excluded.

Since we only had one exclusion criteria and otherwise included all patients undergoing HP in the period September 2016 to August 2021, risk of selection bias was minimal.

### Study variables

The medical charts were examined retrospectively, and data regarding pre-, peri-, and post-operative information was collected using the Research Electronic Data Capture (REDCap).

Preoperative information included sex; age; body mass index (BMI); smoking status; alcohol consumption; indication for HP, T-, N-, and M stages (TNM) if CRC; Hinchey classification if diverticulitis; Charlson Comorbidity Index (CCI); American Society of Anesthesiology (ASA) score; previous radiotherapy in the pelvic area; chemotherapy within 6 weeks; plasma albumin level; previous abdominal surgery; anticoagulant therapy; and prescription of thrombo-embolic deterrent (TED) socks. Alcohol overconsumption was defined as ≥ 7 items per week for women and ≥ 14 items per week for men according to national guidelines [18]. Palpable foot pulses are required to prescribe TED socks at our institution, and prescription of TED socks was used as an indicator of sufficient iliac circulation. Comorbidity was divided into 3 groups according to the CCI-score (0–2 “none/mild,” 3–4 “moderate,” and ≥ 5 “severe”) [19], and BMI values were divided into groups according to the World Health Organization (WHO) [20]. Plasma albumin level was divided into two groups, above and below lower reference value [21].

Surgical information included setting (acute/subacute/elective), duration of surgery, perioperative rectal stump management, supplementary HIPEC, peri- or postoperative blood transfusion, and peritoneal contamination. Surgery undertaken during a planned admission was categorized as elective, surgery within 6 h of admission was considered acute, and surgery within an acute admission (but after 6 h) was considered subacute. At our institution, the rectal remnant is routinely stapled with double or triple staple rows and/or hand sutured (Table S2). A Foley catheter is routinely inserted intraoperatively in the rectal remnant in emergency patients and is typically removed after 1–3 days or until secretion has stopped.

### Outcome measures

The primary outcome was incidence of blowout within 90 days after a primary HP. Blowout was defined as a defect in the resection line of the rectum causing leakage of the rectal remnant verified by computed tomography (CT) or endoscopy within 90 days post-operatively. Pelvic abscesses, diagnosed on CT, were considered a result of leakage from the rectal remnant following blowout and was included as blowout. Contrast was not routinely administered rectally during CT.

Secondary outcomes were potential risk factors for blowout, LOS, readmission rate within 90 days post-operatively, and 30- and 90-day mortalities.

### Statistical analysis

Variables are presented in contingency tables as number and percentage of patients in the blowout and non-blowout group and in total for each variable. Continuous variables were transformed to categorical variables based on relevant groupings or above and below the median value.

Univariate statistical analyses were initially performed on potential predictors for blowout using *Pearson’s chi-square* or *Fischer’s exact* test. Relative risks with 0.95 confidence intervals (CIs) were calculated for dichotomous variables.

Multivariate analysis was performed using binomial logistic regression on the variables associated with blowout in our univariate analyses and potential risk factors for blowout after HP according to literature (sex, poor iliac circulation, previous radiotherapy) [11, 12].

*P* values < 0.05 were considered statistically significant. All calculations were performed using Stata/MP 17.0.

## Results

### Baseline

In total, 220 patients were identified for medical chart review. Forty-two were excluded, leaving 178 patients for further analysis. Exclusion was due to an incorrectly coded surgical procedure (*n* = 29), HP being performed as a secondary surgery (due to complications from primary surgery) (*n* = 10), or resection only orally from an already existing colostomy (*n* = 3).

There were 98 females (55%) and 80 males (45%). The mean age was 65 years (range 18–92). Indications for HP were CRC (45.5%), diverticulitis (24.2%), gynecological cancer (3.9%), and other (26.4%), including pseudomyxoma peritonei, acute bowel ischemia, inflammatory bowel disease, volvulus, bowel perforation, fecal incontinence, and other benign conditions. Among the diverticulitis patients, 4 patients had Hinchey I, 9 patients had Hinchey II, 8 patients had Hinchey III, 12 patients had Hinchey IV, and 10 patients had a HP due to chronic or recurrent diverticulitis.

Supplemental HIPEC along with HP was performed in 58 patients (32.6%) and 8 patients had previously received pelvic radiotherapy. Patient characteristics are presented in Table S1.

In total, 69 patients (38.8%) resided outside Central Denmark Region resulting in limited access to medical charts originating before admission and after discharge from our institution.

### Blowout

During the 90 days of follow-up after HP, 30 patients (16.9%) developed blowout after a median of 12 days (range 5–37) post-surgery. Of these, 14 (46.7%) presented with a defective closure line of the rectum, whereas 16 (53.3%) only had a pelvic abscess and no signs of closure line defect. All cases of blowout had a CT scan, and 11 of the 14 defective closure lines were verified with endoscopy. The three patients with a defect resection line that were not diagnosed with endoscopy were instead diagnosed based on the combination of a pelvic abscess on CT combined with rectal discharge of pus. Blowout characteristics are seen in Table 1.

**Table 1:** Blowout characteristics

Characteristics	*N* (%)
Total number of blowout cases	30/178 (16.85)
CT-verified	30/30 (100)
Defective staple/suture line detected	
No Yes	16/30 (53.33) 14/30 (46.67)
Defect endoscopy-verified Defect diagnose based on pelvic abscess and rectal discharge of pus	11/14 (78.6) 3/14 (21.4)
Days from HP to blowout, median (range)	12 (5–37)

### Predictors of blowout

Blowout within 90 days after a primary HP was significantly associated with Hinchey IV diverticulitis (RR 2.77 (95% CI 1.29, 5.92)) and alcohol overconsumption (RR 2.14 (95% CI 1.03, 4.47)) in univariate analyses. Blowout was not associated with any of the preoperative variables: sex, age, smoking, BMI, radiotherapy, comorbidity status, ordination of TED socks, ASA score, indication for HP, localization, or TNM stage of CRC. Univariate analyses of preoperative variables and blowout are shown in Table S1.

Intraoperative insertion of a rectal Foley catheter was associated with reduced risk of blowout within 90 days after primary HP (RR 0.38 (95% CI 0.14, 1.04)). No other perioperative variable was significantly associated with blowout after primary HP as seen in Table S2.

Multivariate analysis with binomial logistic regression was performed on variables significantly or nearly significantly associated with blowout in our univariate analyses (Hinchey IV, alcohol overconsumption, and intraoperatively inserted rectal Foley catheters) or according to literature (male sex, lacking TED sock ordination as a substitute for lacking foot pulses, and previous radiotherapy). The result is presented in Table 2 and displays increased risk of blowout among patients with Hinchey IV diverticulitis (RR 6.32 (95% CI 4.09–9.75)), previous radiotherapy (RR 3.35 (95% CI 1.67–6.74)), and alcohol overconsumption (RR 1.69 (95% CI 1.05–2.72)). Patients with an intraoperatively inserted rectal Foley catheter had reduced risk of blowout within 90 days after primary HP (RR 0.18 (95% CI 0.05–0.65)). Multivariate analysis showed no association between blowout and sex or ordination of TED socks.

**Table 2:** Evaluation of independent factors for blowout by multivariate analysis (binomial logistic regression)

Variable	Risk ratio	95% CI	*P*
Male	0.73	0.45, 1.17	0.19
Alcohol overconsumption	1.69	1.05, 2.72	0.03
Hinchey IV	6.32	4.09, 9.75	< 0.001
Radiotherapy	3.35	1.67, 6.74	0.001
Lack of TED socks	0.77	0.12, 5.00	0.78
Foley catheter	0.18	0.05, 0.65	0.03

### LOS, readmission, and 30- and 90-day mortality

LOS, readmission rate, and mortality rates are shown in Table 3. The median LOS after primary HP was 12 days (range 1–106) and was longer among blowout than non-blowout patients (21.5 and 10 days, respectively, *p* < 0.0001). Of the 69 patients residing outside Central Denmark Region, 43 patients (24.2% in total) were transferred from our institution to a hospital in another region before final discharge and after a median of 10 days (range 3–75).

**Table 3:** Postoperative data of patients who underwent Hartmann’s procedure

Variable	Total *n* = 178 (%)	Non-blowout *n* = 148 (%)	Blowout *n* = 30 (%)	*p* value^a^ RR (95% CI)
Median LOS (range)	12 (1–106)	10 (1–48)	21.5 (6–106)	< 0.0001
Readmission	43 (24.2)	36 (24.3)	7 (23.3)	0.91
30-day mortality	11 (6.2)	9 (6.1)	2 (6.7)	1.00
90-day mortality	18 (10.1)	12 (8.1)	6 (20.0)	0.049

^a^*P* values for Pearson’s chi-square, Fischer’s exact, Student’s *t* test, Mann–Whitney *U*-test as appropriate

Readmission rate within 90 days after primary HP was 24.2%. Patients were readmitted 12 days (median, range 1–68) after primary discharge. Readmission rate was not associated with blowout (*p* = 0.908). It was, however, higher among blowout patients with defective closure lines (85.7%) compared to blowout patients with intact closure lines and pelvic abscess only (14.3%) (*p* = 0.026). Other reasons for readmission were constipation, ileus, fever, vomiting, and abdominal pain, among others.

The overall 30-day mortality rate was 6.2% (11/178) and did not differ significantly between groups (*p* = 0.903). The overall 90-day mortality rate was 10.1% (18/178) and was significantly higher in the blowout group compared to the non-blowout group (20.0% and 8.1%, respectively, *p* = 0.049).

## Discussion

We found an incidence of 16.9% of blowout within 90 days after a primary HP, of which almost half had a defect in the resection line of the rectal remnant. This is consistent with historic studies reporting a 17% pelvic abscess rate [12, 13] after HP, although a study from 2018 has reported a pelvic sepsis rate (i.e., pelvic abscesses or rectal secretion of pus) of only 6.4% [11]. The latter study led to the assumption that pelvic sepsis was a rather unusual event following HP and that HP was safer than previously anticipated [11].

The observed discrepancy in the literature might be caused by different patient cohorts being examined, potentially leading to pronounced variation in patient characteristics between studies, especially considering the various indications for HP. Also, follow-up periods vary between 90 days in our study and over 300 days in others [11, 12]. This may have underestimated the blowout rate in our study compared to others; however, blowout occurred within 37 days in our study.

Nonetheless, according to our findings, blowout should once again be considered a frequent complication following a primary HP.

Patients with Hinchey IV diverticulitis, alcohol overconsumption, or previous pelvic radiotherapy were found to be at increased risk of blowout according to our multivariate analysis. Hinchey IV diverticulitis and radiotherapy have previously been reported as risk factors for pelvic abscess formation following HP [11]. We found a more than 6- and 3-times increased risk of blowout after a primary HP among patients with Hinchey IV and previous pelvic radiotherapy, respectively. This is not surprising as both induce pelvic inflammation impairing the conditions for wound healing, facilitating closure line dehiscence in the rectal remnant and formation of pelvic abscesses. However, we cannot exclude that other factors; e.g., severity grade of septicemia might increase the risk of rectal blowout rather than just Hinchey IV diverticulitis specifically.

Alcohol overconsumption and peritoneal contamination (e.g., Hinchey IV) are known risk factors for anastomotic leakage [22–24], indicating a shared pathophysiology.

An intraoperatively inserted rectal Foley catheter was associated with 82% reduced risk of blowout within 90 days after a primary HP in our multivariate analysis. Foley catheters secure drainage of the rectal remnant in case of retained mucus and fluids; however, they are mostly used in patients undergoing emergency surgery. With our findings, we recommend considering intraoperative insertion of a rectal Foley catheter in all patients undergoing a primary HP, if technically possible, regardless of surgical setting, especially since this is a minimally invasive and low-cost procedure.

Considering the high blowout rate, selecting the surgical procedure should be done with caution, and surgical alternatives to a primary HP should be considered. A recent prospective cohort study has demonstrated a lower pelvic abscess rate in patients undergoing intersphincteric abdominoperineal excision (iAPE) compared to HP [14], and there is currently an ongoing randomized controlled trial where iAPE and HP are compared [25].

Previous studies have further identified male sex, lack of foot pulses, and rectal resection ≤ 2 cm above the pelvic floor as potential risk factors for blowout after HP [11, 12]. We were unable to reproduce this in our study since sufficient details regarding resection level were lacking in the medical charts. Tøttrup et al. [12] found lack of palpable foot pulses as an indicator of poor iliac circulation to be associated with blowout following low HP, indicating that relative ischemia in the rectal remnant could be a contributing factor to resection line defect. The fact that we used TED socks as a substitute for palpable foot pulses might have underestimated the association with blowout, as poor iliac circulation indicated by lack of foot pulses might not have been the only reason for patients not having TED socks prescribed. Of importance, lack of foot pulses might be less associated with blowout after a high HP, since a longer rectal stump receives its blood supply not only from the iliac arteries.

Patients developed blowout after a median of 12 days compared to 35 days in a similar study from our institution, published in 2005 by Tøttrup et al. [12], which might indicate that blowout is diagnosed in an earlier stage today. All blowout patients in our study had a CT scan performed, whereas only 38.7% of the pelvic abscesses were diagnosed with CT scan in the previous study. Of the patients with detected pelvic abscesses in the study by Tøttrup et al., 87% (27/31) also had a defect staple line, whereas only 47% (14/30) of blowout patients in our study had a defect staple line. Therefore, the use of CT imaging should continue being liberal so that pelvic abscesses with milder symptoms are detected in as early a stage as possible.

The hospital stay was significantly longer, and the 90-day mortality rate was significantly higher among the patients that developed blowout after HP. A recent study found no difference in LOS between blowout and non-blowout patients [11] and two studies [11, 12] reported no mortality in the blowout groups. We found a 90-day mortality rate of 20% among blowout patients compared to 8.1% among non-blowout patients demonstrating that blowout is a serious complication in these patients.

Few studies have investigated risk factors for blowout after HP, and, to our knowledge, multivariate analysis adjusting for potential confounders has not previously been performed. However, given the retrospective design of our study, some variables could not be consistently recorded for all patients. Missing data was limited except for information regarding preoperative albumin level (18 missing) and ordination of TED socks (13 missing). However, these were evenly distributed among patients with blowout and no blowout. Unfortunately, sufficient data on resection level was lacking. Thus, we were unable to evaluate this variable as a potential risk factor for blowout.

The total number of patients developing blowout after a primary HP might have been underestimated among the patients residing outside Central Denmark Region, as our access to the medical charts ceased when these patients were discharged from our institution either directly to their home or transferred to hospitals outside Central Denmark Region. However, patients were only transferred from our institution when recovering well.

Furthermore, some patients underwent supplemental surgery during the same procedure as HP, e.g., pelvic exenteration, other bowel resection, and splenectomy, which we did not record as individual study variables. This might have contributed to bias of our primary endpoint due to potentially increased surgical stress response in these patients, which may have affected the incidence of blowout within 90 days. Additionally, we cannot exclude that some pelvic abscesses were a result of other conditions rather than blowout.

## Conclusion

According to our findings, blowout occurs in 16.9% of patients within 90 days after primary surgery with Hartmann’s procedure and thus remains a common post-operative complication. HP should therefore be considered with caution especially among selected high-risk groups. Hinchey IV diverticulitis, radiotherapy in the pelvic area, and alcohol overconsumption each increase the risk of developing blowout within 90 days after a primary HP. Patients developing blowout after a primary HP have longer hospital stays and higher 90-day mortality, emphasizing the severity of blowout. Intraoperative insertion of a Foley catheter in the rectal remnant might reduce the risk of blowout considerably and should be considered used more liberally in all patients undergoing a primary HP.

### Supplementary Information

Below is the link to the electronic supplementary material.

Supplementary file1 (DOCX 20 KB)

Supplementary file2 (DOCX 17 KB)
