# Supplementary material for: Incidence and risk factors of blowout within 90 days after a primary Hartmann’s procedure: a retrospective cohort study
Source: Langenbecks Arch Surg. 2023 Jul 14;408(1):275. doi: 10.1007/s00423-023-02967-5 (PMC10345077; doi:10.1007/s00423-023-02967-5)
Supplement: Supplementary file 1 — Supplementary file1 (DOCX 20 KB) [file 423_2023_2967_MOESM1_ESM.docx]

### **Table 1: Demographic data and blowout of patients who underwent Hartmann’s procedure**

| Variable | Total  n = 178 (%) | Non-blowout  n = 148 (%) | Blowout  n = 30 (%) | RR  (95% CI) | p-value^a^ |
| --- | --- | --- | --- | --- | --- |
| Sex  Female  Male | 98 (55.1)  80 (44.9) | 82 (55.4)  66 (44.6) | 16 (53.3)  14 (46.7) | 1.07 (0.56,2.06) | 0.84 |
| Age  ≤ 67 years  > 67 years | 91 (51.1)  87 (48.9) | 77 (52.0)  71 (48.0) | 14 (46.7)  16 (53.3) | 1.20  (0.62,2.30) | 0.59 |
| Smoking status  Never  Previous  Current  Missing | 69 (38.8)  60 (33.7)  47 (26.4)  2 (1.1) | 60 (40.5)  47 (31.8)  39 (26.4)  2 (1.3) | 9 (30.0)  13 (43.3)  8 (26.7)  0 | 1.50 (0.73,3.09) | 0.43 |
| Alcohol  No overconsumption  Overconsumption  Missing | 148 (83.2)  23 (12.9)  7 (3.9) | 127 (85.8)  16 (10.8)  5 (3.4) | 21 (70.0)  7 (23.3)  2 (6.7) | 2.14 (1.03,4.47) | 0.05 |
| BMI  <18.5  18.5-25  25-30  ≥30  Missing | 13 (7.3)  61 (34.3)  68 (38.2)  35 (19.7)  1 (0.6) | 11 (7.4)  50 (33.8)  57 (38.5)  29 (19.6)  1 (0.7) | 2 (6.7)  11 (36.7)  11 (36.7)  6 (20.0)  0 |  | 0.99 |
| Indication of surgery  CRC  Gyn. cancer  Diverticulitis  Other | 81 (45.5)  7 (3.9)  43 (24.2)  47 (26.4) | 66 (44.6)  5 (3.38)  35 (23.7)  42 (28.4) | 15 (50.0)  2 (6.7)  8 (26.7)  5 (16.7) |  | 0.43 |
| Hinchey IV  No Hinchey IV | 12 (6.7)  166 (93.3) | 7 (4.7)  141 (95.3) | 5 (16.7)  25 (83.3) | 2.77  (1.29,5.92) | 0.02 |
| p-Albumin (g/L)  ≥36  <36  Missing | 55 (30.9)  105 (59.0)  18 (10.1) | 48 (32.4)  85 (57.4)  15 (10.1) | 7 (23.3)  20 (66.7)  3 (10.0) | 1.50  (0.67,3.32) | 0.31 |
| Comorbidity  None/mild  Moderate  Severe | 77 (43.3)  21 (11.8)  80 (44.9) | 63 (42.6)  17 (11.5)  68 (46.0) | 14 (46.7)  4 (13.3)  12 (40.0) |  | 0.81 |
| ASA-score  ASA I-II  ASA III-IV | 111 (62.4)  67 (37.6) | 91 (61.5)  57 (38.5) | 20 (66.7)  10 (33.3) | 0.83  (0.41,1.66) | 0.59 |
| Preoperative chemotherapy within 6 weeks  No  Yes | 161 (90.5)  17 (9.6) | 131 (88.5)  17 (11.5) | 30 (100)  0 | 0 | 0.08 |
| Previous radiotherapy in pelvic area  No  Yes | 170 (95.5)  8 (4.5) | 143 (96.6)  5 (3.4) | 27 (90.0)  3 (10.0) | 2.36 (0.90,6.16) | 0.13 |
| Lack of TED  No  Yes  Missing | 152 (85.4)  13 (7.3)  13 (7.3) | 125 (84.5)  12 (8.1)  11 (7.4) | 27 (90.0)  1 (3.3)  2 (6.7) | 0.43 (0.06,2.94) | 0.70 |

^a)^P-values for Pearson’s chi square or Fischer’s exact as appropriate
