# Supplementary material for: Incidence and risk factors of blowout within 90 days after a primary Hartmann’s procedure: a retrospective cohort study
Source: Langenbecks Arch Surg. 2023 Jul 14;408(1):275. doi: 10.1007/s00423-023-02967-5 (PMC10345077; doi:10.1007/s00423-023-02967-5)
Supplement: Supplementary file 2 — Supplementary file2 (DOCX 17 KB) [file 423_2023_2967_MOESM2_ESM.docx]

**Table 3: Perioperative data and blowout of patients who underwent Hartmann’s procedure**

| Variable | Total  n = 178 (%) | Non-blowout  n = 148 (%) | Blowout  n = 30 (%) | RR  (95% CI) | p-value^a^ |
| --- | --- | --- | --- | --- | --- |
| Setting  Elective  Acute/subacute | 106 (59.6)  72 (40.5) | 89 (60.1)  59 (39.9) | 17 (56.7)  13 (43.3) | 1.13  (0.58,2.17) | 0.72 |
| Duration of surgery  ≤ 196 min  > 196 min  Missing | 88 (49.4)  88 (49.4)  2 (1.1) | 71 (48.0)  75 (50.7)  2 (1.3) | 17 (56.7)  13 (43.3)  0 | 0.76  (0.40,1.48) | 0.42 |
| Rectal stump irrigation  No  Yes | 158 (88.8)  20 (11.2) | 130 (87.8)  18 (12.2) | 28 (93.3)  2 (6.7) | 0.56 (0.15,2.19) | 0.53 |
| Type of rectal stump closure  Stapled  Hand sutured  Both  Missing | 149 (83.7)  3 (1.7)  5 (2.8)  21 (11.8) | 124 (83.8)  1 (0.7)  4 (2.7)  19 (12.8) | 25 (83.3)  2 (6.7)  1 (3.3)  2 (6.7) |  | 0.119 |
| Foley catheter in rectum  No  Yes | 127 (71.4)  51 (28.7) | 101 (68.2)  47 (31.8) | 26 (86.7))  4 (13.3) | 0.38 (0.14,1.04) | 0.047 |
| HIPEC  No  Yes | 120 (67.4)  58 (32.6) | 101 (68.2)  47 (31.8) | 19 (63.3)  11 (36.7) | 1.20 (0.61,2.35) | 0.60 |
| Peri-/postoperative blood transfusion  No  Yes | 97 (54.5)  81 (45.5) | 84 (56.8)  64 (43.2) | 13 (43.3)  17 (56.7) | 1.57 (0.81,3.03) | 0.18 |

^a)^P-values for Pearson’s chi square and Fischer’s exact test, as appropriate
